# Education Research: Strengthening the Neurology Pipeline: The American Academy of Neurology Medical Student Symposium (2018–2025)

**DOI:** 10.1212/NE9.0000000000200373

**Published:** 2026-11-02

**Authors:** Katherine A. Fu, Christy J. Soares, Justin T. Jordan, Ria Geguera, Michael Urvig, Jose Posas, Aaron S. Zelikovich, Gregory S. Day, Rana R. Said

**Affiliations:** 1 Department of Neurology, University of California, Los Angeles;; 2 Department of Neurology, Emory University, Atlanta, GA;; 3 Department of Neurology, University of Texas Southwestern Medical Center, Dallas;; 4 Workforce Development, American Academy of Neurology, Minneapolis, MN;; 5 Member Insights, American Academy of Neurology, Minneapolis, MN;; 6 Department of Neurology, University of Virginia, Charlottesville;; 7 Department of Neurology, Zucker School of Medicine, New York, NY;; 8 Department of Neurology, Mayo Clinic in Florida, Jacksonville; and; 9 Division of Pediatric Neurology, Department of Pediatrics, University of Texas Southwestern Medical Center, Dallas.

## Abstract

**Background and Objectives:**

The American Academy of Neurology (AAN) Medical Student Symposium (MSS) was established in 2018 to address the shortage of neurologists, with events consisting of keynote speakers, panel events, and networking opportunities to engage medical students with neurology. The aim of this study was to highlight the evolution of the MSS, to identify the elements participants perceived most beneficial, and to describe the longitudinal impact of the symposium on attendees.

**Methods:**

We describe the MSS, recruitment processes, and its evolution from 2018 to 2025. To determine the longitudinal impact of the MSS on medical student interest in neurology, 1,177 attendees of past MSS were invited to participate in an online survey. Data were collected using Qualtrics from August 25, 2025, to September 19, 2025.

**Results:**

MSS student attendance between 2018 and 2025 grew from 152 in 2018 to 353 participants in 2025. Most attendees were third-year medical students (MS3, 34%), followed by second-year (MS2, 19%) and fourth-year (MS4, 21%) medical students. In total, 129/1,079 individuals responded, and 120/129 respondents completed the survey. Interest in a neurology specialty increased after the MSS for 92% of participants. The components ranked as most influential included the networking lunch and subspecialty networking. As of 2025, 39% of 2018 MSS attendees were active AAN members, whereas 82% of 2025 attendees were active members.

**Discussion:**

Most participants expressed increased interest in neurology after the MSS, reporting that the networking components of the event were most influential. Participants highlighted increased knowledge of neurologic careers and opportunities for mentorship as the most impactful elements of the MSS. Longitudinal survey results indicated sustained interest in careers in neurology and continued AAN engagement. Study limitations include possible selection and response bias. Given the lack of a distinct comparison group, we are also unable to assess the MSS's impact on those with neutral or little preexisting interest in neurology. Future MSS iterations will continue to streamline the evaluation process to collect attendee feedback and adapt the agenda based on participant needs.

## Introduction

As the population continues to age, demand for neurologists is expected to grow in-step with increased numbers of age-related neurologic conditions, such as stroke and neurodegenerative disease.^1^ Already, there are critical shortages in rural areas and areas with greater numbers of uninsured patients.^2^

Expanding the neurology pathway and encouraging the development of neurology clinicians has served as one of multiple proposed solutions to address this shortage. Factors shown to support early interest in neurology careers include early and broad exposure to career options in neurology, a strong preclinical neuroscience curricular experience, and interactions with neurology providers and longitudinal mentorship relationships.^3^ To support opportunities in these areas, extracurricular career events to introduce medical students to the range of diverse careers in neurology have been held at academic centers. Events such as “Neuro Day” schedule first-year medical students to rotate through stations of patient-educators with real neurologic diseases paired with physician-educators to moderate the discussion.^4,5^ Hands-on procedure workshops offering opportunities for medical students to practice lumbar punctures, learn about botulinum toxin injections, and neuromodulation devices have also been implemented to spur interest in the field.^6^ Similar hands-on workshops have been implemented to spur interest in other fields and disciplines, primarily in surgical subspecialties, with events involving simulations, cadaver laboratories, surgical skills workshops, and operative videos.^7-9^ Some of these events and conferences have also incorporated elements such as career seminars, keynote speakers, poster symposia, and networking events to allow medical students to establish connections with faculty and other trainees in the field to foster a greater understanding of careers in these fields, work-life balance, and the culture of the discipline.^7,10^

Akin to these efforts, the American Academy of Neurology (AAN) Medical Student Symposium (MSS) was established in 2018 to support the neurology pathway and address the shortage of neurology clinicians by promoting interest in careers in neurology. The Symposium was supported by funding from the Conrad N. Hilton Foundation, granted to address the growing shortage of neurologists by encouraging medical students to enter the field of neurology. Development and organization of the MSS were led by the AAN Pipeline Subcommittee. Here, this study aimed to highlight the goals of the MSS and the iterative improvements to the symposium from inception (2018) to 2025. We showcase key lessons learned as pertains to event organization for programs interested in adapting elements of the symposium to similar events at their institution. We also report the components that have been found to be most meaningful to medical students in inspiring their pursuit of careers in neurology and potential indicators of the long-term impact of the MSS on attendees. These lessons can inform future endeavors across educational programs within neurology, allowing programs to consider how best to leverage the institutional resources available and maximize their impact among their medical students.

## Methods

### Medical Student Symposium Overview

The MSS is a half-day event held in-person at the AAN Annual Meeting. The annual MSS program has evolved over the years, including a temporary shift to a virtual format in 2020–2021 due to the COVID-19 pandemic, with various agenda items consisting of: (1) an introductory lunch with small groups of students, a neurology attending, and a neurology resident or fellow at each table; (2) lecture or panel-style programming to highlight advances in neurology, career paths in neurology, advocacy in neurology, and global neurology, among other topics; (3) interactive sessions such as hands-on exploration of medical devices relevant to the practice of neurology, as well as speed-dating style engagement with attending neurologists to learn about their subspecialties; and (4) refreshments and informal networking among student participants, neurology attendings, and resident physicians.

### Recruitment

The AAN offers multiple student scholarships to defray the costs of attending the Annual Meeting, which cover air travel, hotel, and registration.^11^ Each scholarship has a tailored program with required and recommended programming and is offered to no more than one student per program. Scholarships are publicized on the AAN website, through AAN trainee specific e-newsletter, by word of mouth from educators, and through social media platforms. Two exemplary scholarship programs are the Medical Student Scholarship to the Annual Meeting (formerly Medical Student Experience at the Annual Meeting Scholarship, with 50 recipients being first-year and second-year medical students) and the Medical Student Diversity Scholarship to the Annual Meeting (10 recipients, targeted to second-year and third-year students). Students apply for the scholarship that best fits their interests or career stage, are scored by a review committee, and are selected for the scholarship based on application score. Of the medical students who attend the Annual Meeting, around 130 each year are scholarship recipients. Medical student scholarship recipients are required to attend the MSS as a part of their meeting experience, thus ensuring a broad audience for the program. Recipients experience dedicated educational activities, scientific presentations from leaders in the field of neurology, and opportunities to enrich their medical careers and broaden their professional networks.

### Medical Student Symposium Evolution (2018–2025)

Registration was requested before the Annual Meeting, but beginning in 2021, individuals who did not pre-register were able to walk in and participate.

The initial emphasis of the MSS when launched in 2018 was to address the phenomenon of “neurophobia,”^12^ offering events through the symposia to demonstrate to students the relevance of neurology and neuroscience in pop culture as well as introduce students to neurology trainees and attendings practicing in the field. This began with a welcome lunch for medical students to engage with a neurology attending physician and resident or fellow, followed by a 1-hour round-robin networking session where medical students were able to meet attending neurologists from a wide variety of subspecialties in 15-minute increments. Medical students then participated in a “Neurology in Pop Culture” lecture. The final portion of the symposium was a mixer with refreshments to network with faculty and other trainees. The 2019 MSS had a similar format to 2018 with the addition of a session that involved demonstrations of neurologic technology and procedures including EEG, EMG, DBS, botulinum toxin injections, and interarterial clot removal. These hands-on demonstrations were added in response to early attendee feedback reflecting enthusiasm for practical exposure to neurologic procedures and technology and with the aim of countering the perception that neurology lacks procedural breadth. Unfortunately, owing to the COVID-19 pandemic, the 2020 symposium was canceled.

After the COVID-19 pandemic, the neurology community experienced a renewed emphasis on scientific innovation and global health equity. The 2021 symposium was accordingly redesigned around these themes, offering a virtual format that included a session on “Innovations in Neurology” to highlight advances in neurologic therapeutics and devices, followed by “Neurology in Action,” which explored advocacy opportunities spanning local to global community levels. The virtual format, while necessitated by the pandemic, also allowed for broader geographic participation from students who would not otherwise have been able to attend the Annual Meeting. In 2022, the emphasis on innovation in neurology persisted with the in-person return of the Annual Meeting, which enabled the reintroduction of live networking sessions—a format identified in earlier years as highly valued by participants. Two innovation sessions on gene therapy and technological innovations were offered alongside a subspecialty networking session and networking mixer.

In 2023, the symposium began with a welcome lunch for medical students, followed by a 30-minute keynote lecture on the “multiverse” in neurology, with mention of the many career opportunities in neurology while alluding to the “multiverse” references in pop culture at the time. This was followed by a panel discussion on the pathways of neurology through residency, fellowship, and into careers in neurology. The second half of the symposium featured networking with trainees and neurologists from various subspecialties. The 2023 agenda represented a deliberate return to career-focused programming, with the multiverse framing selected to engage students through a culturally resonant lens while conveying the breadth of neurologic career paths. The reintroduction of the welcome lunch reflected feedback from prior years indicating that small-group, informal interactions with faculty and trainees were among the most meaningful components of the event. In 2024, the main events shifted to the addition of a second panel in lieu of a keynote speaker based directly on participant feedback from the 2023 MSS, in which attendees expressed interest in hearing multiple perspectives on the neurologist's experience and career path rather than a single presenter's viewpoint. The 2 panels' discussion topics were “the life of a neurologist” and “the path to becoming a neurologist.” The panel sessions were followed by a subspecialty networking session. In 2025, the welcome lunch with faculty and residents/fellows was intentionally lengthened based on cumulative evidence from postevent evaluations and the longitudinal survey that small-group, relationship-centered interactions were the most influential components of the MSS for participants, and followed by a single panel discussion on “journeys in neurology.” The second half of the symposium was a subspecialty networking session. The evolution of the MSS agenda is summarized in a Sankey Diagram format (Figure 1).

**Figure 1:**
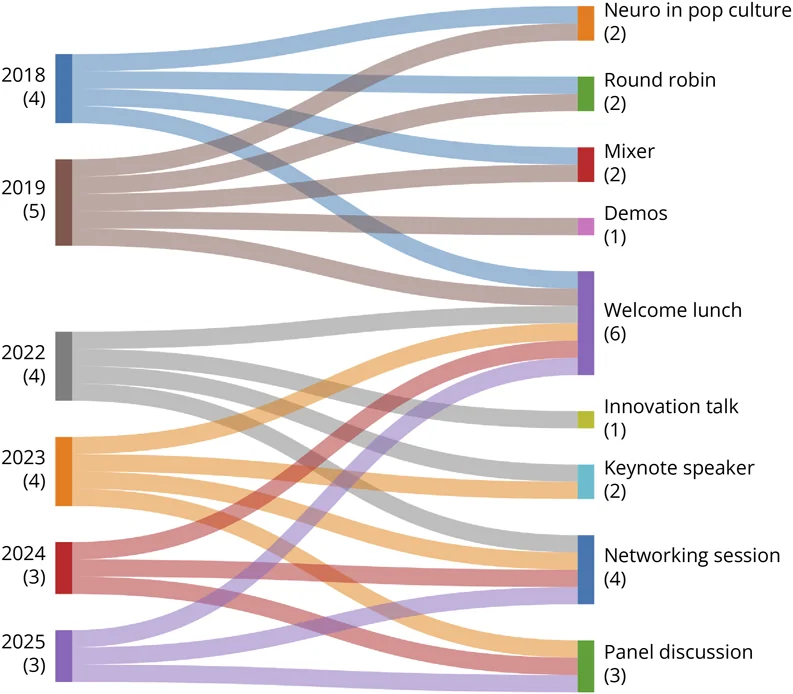
Sankey Diagram Highlighting Key Events and the Evolution of the Medical Student Symposium From 2018 to 2025 Note that 2020–2021 were omitted due to impact from the COVID-19 pandemic. Numbers indicate the total number of events. For example, the 4 events in 2018 were “Neuro in Pop Culture,” “Round Robin,” “Mixer,” and “Welcome Lunch.” The Neuro in Pop Culture event was held twice: once in 2018 and again in 2019.

### Medical Student Attendance Analysis

For statistical analysis, data pertaining to the total number of medical student attendees were gathered annually through registration forms, and starting in 2023, walk-ins were also tracked. The overall participation was then compared as a total and then separated by year of training. The difference in these data was analyzed using compound annual growth rate to assess overall growth from 2018 to 2025.

#### Trainee/Faculty Volunteers Attendance Analysis

Data pertaining to the total number of trainee and faculty volunteers were gathered through internal documents organized by AAN staff and event organizers. We quantified the number of resident, fellow, and faculty volunteers, including how many returned in a subsequent year.

### Medical Student Symposium Evaluation

The evolution of the MSS agenda was influenced by a combination of priorities of the AAN Education Community and by the annual feedback received from medical student participants as well as volunteers. Over the course of the symposium's implementation, evaluation approaches also evolved. In 2018 and 2019, attendees were asked to complete a 10-question survey with 3 additional open-ended questions at the completion of the event. Formal evaluations were not collected during 2020 as the MSS was canceled and in 2021 because of the virtual format of the MSS during the COVID-19 pandemic. The MSS evaluation responses were limited in 2022–2023 because of a low response rate. The 2023 MSS strove to address this issue by distributing anonymous links through email to medical student attendees and faculty volunteers after the event, the responses which were then analyzed and contributed to the changes in the agenda for 2024.

During 2024–2025, the evaluation process was streamlined to use a single QR code, which would direct respondents to a role-specific evaluation depending on if they were a medical student attendee or faculty/trainee volunteer. Evidence of evaluation completion was necessary for attendees to receive appreciation gifts (i.e., reflex hammer and tuning fork), for participating in the MSS and scholarship recipient reimbursement was similarly contingent on evaluation completion. To incentivize faculty volunteers, a certificate of appreciation from the AAN was distributed with the evaluation.

### Longitudinal Survey Methods

#### Survey Instrument

To determine the longitudinal impact of the MSS on medical student interest in neurology as a career, the AAN MSS Manuscript Workgroup administered a survey to medical student attendees of the MSS. Questions were developed in collaboration between AAN Staff and the AAN Medical Student Symposium Manuscript Workgroup. Key items the survey assessed include year in medical school the participant attended the MSS, current career stage, intended or current specialty and subspecialty, impact of the MSS on interest in a neurologic specialty and subspecialty, ranking components of the MSS, and an open-ended question asking how attending the MSS has affected their career trajectory.

#### Standard Protocol Approvals, Registrations, and Patient Consents

This study (IRB-25-1368) was reviewed by the Institutional Review Board (IRB) of University of California, Los Angeles, and granted certified exempt status.

#### Population

The survey link was emailed to 1,177 attendees of past MSS, constituting a convenience sample based on availability of contact information. The attendee list is maintained by the AAN's Workforce Development team. There were no attendees documented from 2020 or 2021 because no MSS occurred in 2020 and a modified virtual only MSS occurred in 2021 not equivalent to the current or prior iterations of the MSS.

#### Data Collection

Data were collected online using Qualtrics from August 25, 2025, to September 19, 2025. The initial invitation emails were sent on August 25, 2025, with reminder emails to nonrespondents sent on August 27, September 2, and September 15. During distribution, 98 emails were undelivered, resulting in a population of 1,079.

#### Survey Data Analysis

Basic frequencies and summary statistics were conducted using SPSS version 29. Pearson chi-squared tests were used to compare response rates. An inductive top-down coding approach was used for coding open-ended questions using NVivo 15.

### Data Availability

Anonymized survey and evaluation data will be made available by request from qualified investigators. The data will be available for 5 years.

## Results

### Medical Student Symposium Attendance

MSS attendance grew by almost 16% from 2018 to 2019 before the COVID-19 pandemic led to cancellation of the meeting in 2020. A virtual symposium was offered in 2021. The AAN Annual Meeting reconvened in-person in 2022 under the banner of “The Great Neuro Reunion,” with 178 students attending the MSS, matching pre-COVID attendance. There was an increase in attendance from 2022 to 2023 from 178 to 222 registrants. In 2025, there was record attendance of 348 students with a positive compound annual growth rate of 0.11 (Figure 2).

**Figure 2:**
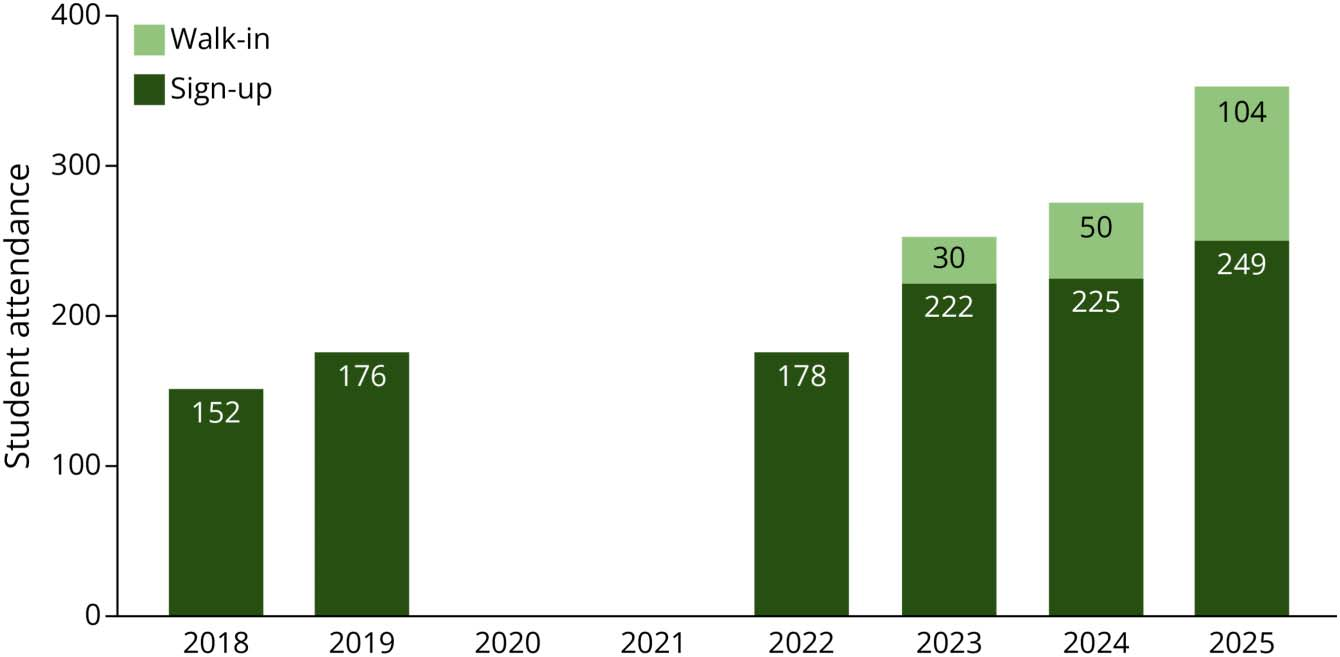
MSS Total Student Attendance Between 2018 and 2025 The 2020 MSS was canceled due to the COVID-19 pandemic, and the 2021 MSS was a virtual-only format; thus, no formal attendance metrics were recorded. MSS = medical student symposium.

Overall, attendance has been predominantly third-year medical students, making up around 34% of the total attendance, with almost equal representation of second-year and fourth-year medical students at 18.9% and 21.0%, respectively. The additional 19.9% of attendees comprised international medical graduates, PhD students, and otherwise not specified. First-year medical students comprised only 5.5% of the total attendance. It was felt that attendance increased from year to year as we adopted a strategy of allowing and encouraging all medical student attendees to the annual meeting to attend, regardless of scholarship status or pre-registration. We also continually sought to improve the MSS and its programming each year.

### Trainee/Faculty Volunteers

Over the course of the MSS, 178 faculty members have volunteered for the event, and 84 faculty (47%) returned a subsequent year. Eighty residents or fellows volunteered, and 29 (36%) returned a subsequent year, with an additional 8 returning to volunteer after having become early career neurologists and faculty themselves. In the evaluations from the 2025 MSS, the most common reasons why volunteers chose to participate in the symposium were because they enjoy talking to potential neurology trainees (87%, n = 40), to share their experience in neurology (78%, n = 36), to serve as a mentor (63%, n = 29), to network with medical students (43%, n = 20), and because they were a past participant of the symposium (20%, n = 9).

### Longitudinal Survey Results

In total, 129/1,079 individuals responded (response rate, 12%), and 120/129 respondents who completed the survey (completion rate, 93%). This response rate is consistent with those reported in similar longitudinal survey-based studies of medical education program participants. Respondents were overrepresented by medical students and underrepresented by AAN former members. No differences in response rate were identified by age or international medical graduate (IMG) status.

Around half of survey respondents indicated attending the MSS as an MS3 (47%), followed by MS4 (27%) and MS2 (23%) (Figure 3A). When asked their current career status, half of participants reported they were a resident, fellow, or practicing physician (50%), with the other half being current or incoming medical students (50%) (Figure 3B). No respondents reported having left medicine. Most participants were not IMG (85%).

**Figure 3:**
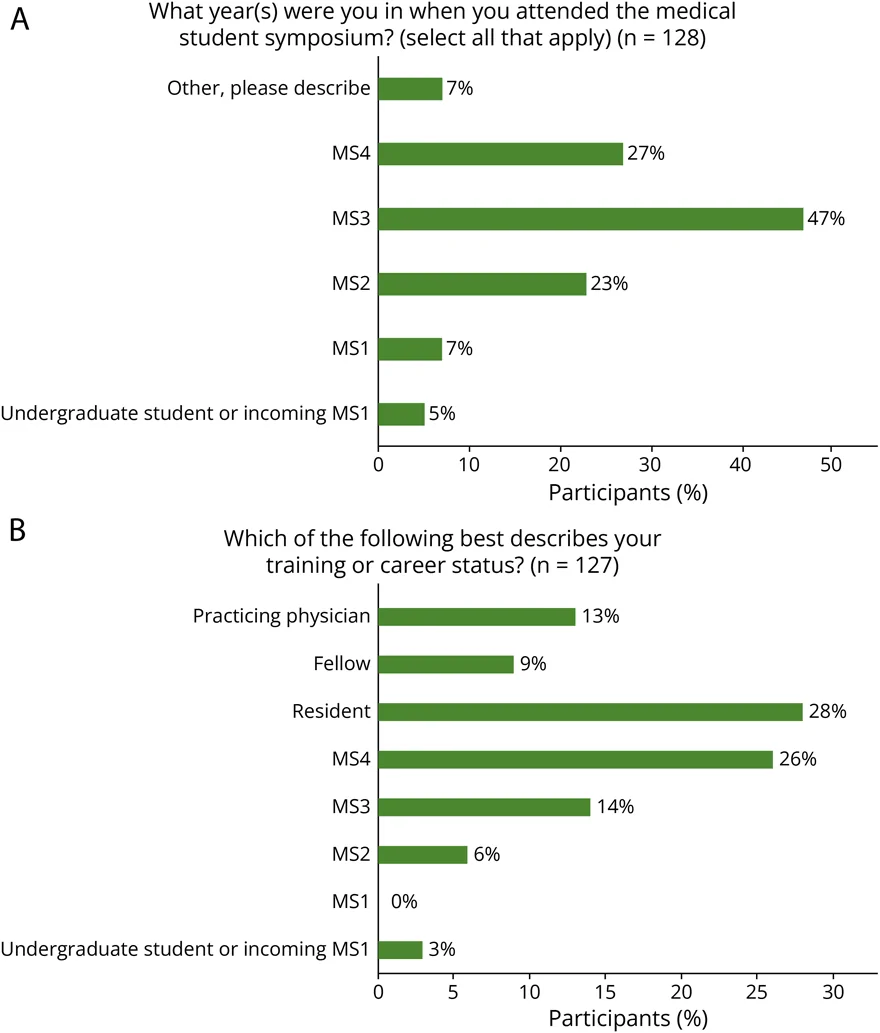
Longitudinal Survey Demographic Responses (A) Respondents' year in medical school at the time of attending the MSS. (B) Description of training or career status at the time of survey completion. MSS = medical student symposium.

Most participants reported intending to pursue a neurology specialty before the MSS with 93% of students, 98% of residents and fellows, and 94% of practicing physicians. Other write-in responses included internal medicine, neurosurgery, and otolaryngology (Figure 4A). Interest in a neurology specialty greatly increased after the MSS for just over half of participants (60%) and somewhat increased for 32%. No participants' interest in a neurology specialty decreased (Figure 4B). Participants were asked what their current intended specialty or current specialty was. Most participants reported a neurology specialty with 89% of students, 87% of residents and fellows, and 100% of practicing physicians. Although no practicing physicians selected other specialties, 11% of students and 13% of residents and fellows indicated other specialties (Figure 4C). Participants were then asked their current intended subspecialty or current subspecialty. The most reported subspecialties included neuroimmunology and multiple sclerosis (n = 13), vascular neurology and stroke (n = 12), neurocritical care (n = 12), and movement disorders (n = 12) (Figure 4D).

**Figure 4:**
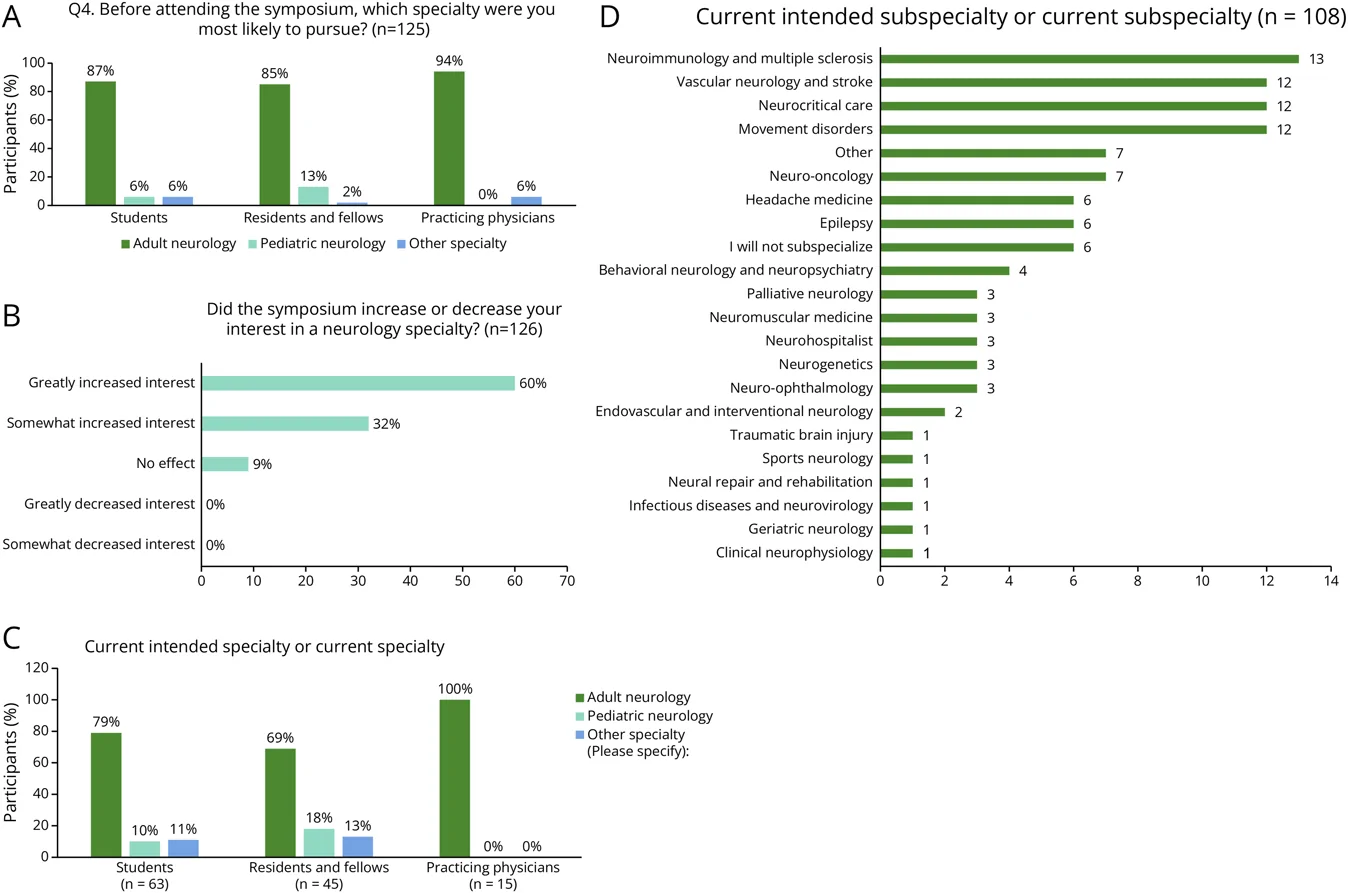
Survey Respondents Indicated (A) The specialty they were most likely to pursue before attending the MSS according to training/career status. (B) Interest in a neurology specialty after attendance of the MSS. (C) Current intended specialty or current specialty. (D) Current intended subspecialty or current subspecialty. MSS = medical student symposium.

Participants were asked to rank different components of the MSS based on their influence on the participant, with 1 indicating the highest influence and 5 the lowest. The Welcome Networking lunch and Subspecialty Networking had similar rankings with both components having a mean rank of 2.0, followed by panel discussion (2.9), keynote speaker (3.3), and other (4.9). Most of the write-in responses for the “other” component reflected events that occurred at the Annual Meeting but not as a part of the MSS (Figure 5).

**Figure 5:**
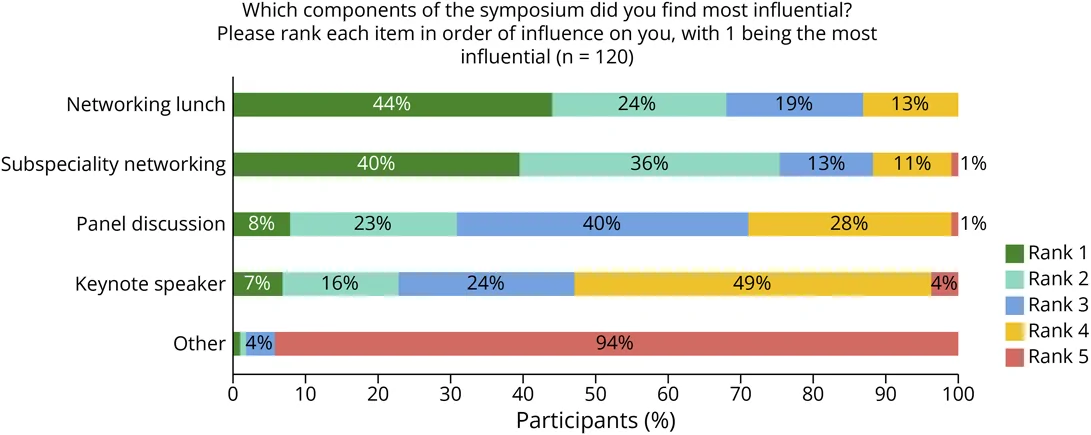
Ranking of Different Components of the MSS Based on Their Influence on the Participant MSS = medical student symposium.

In analyzing the free text responses, when participants were asked how attending the symposium affected their career trajectory, the most common themes mentioned included increased knowledge about a neurology career with frequent references to having learned more about different subspecialties, an increased interest or confirmation of interest in a neurology career, and references to having used the symposium to find their mentor and as a networking opportunity. Some participants also referenced residency applications being helped by attending the symposium by way through the themes of networking, mentors, or increased knowledge.

### Attendees' Continued Engagement With the AAN

Just over a third of 2018 MSS attendees are active AAN members in 2025 (39%) which steadily climbed, with 82% of 2025 attendees being active members in 2025 (Figure 6A). Apart from 2019, between 11% and 15% of MSS attendees went on to attend a subsequent MSS. In 2019, only 2% of attendees attended a subsequent MSS (Figure 6B). The percentage of attendees who went on and attended a subsequent AAN Annual Meeting ranged from 58% in 2018 to 30% in 2024 (Figure 6C).

**Figure 6:**
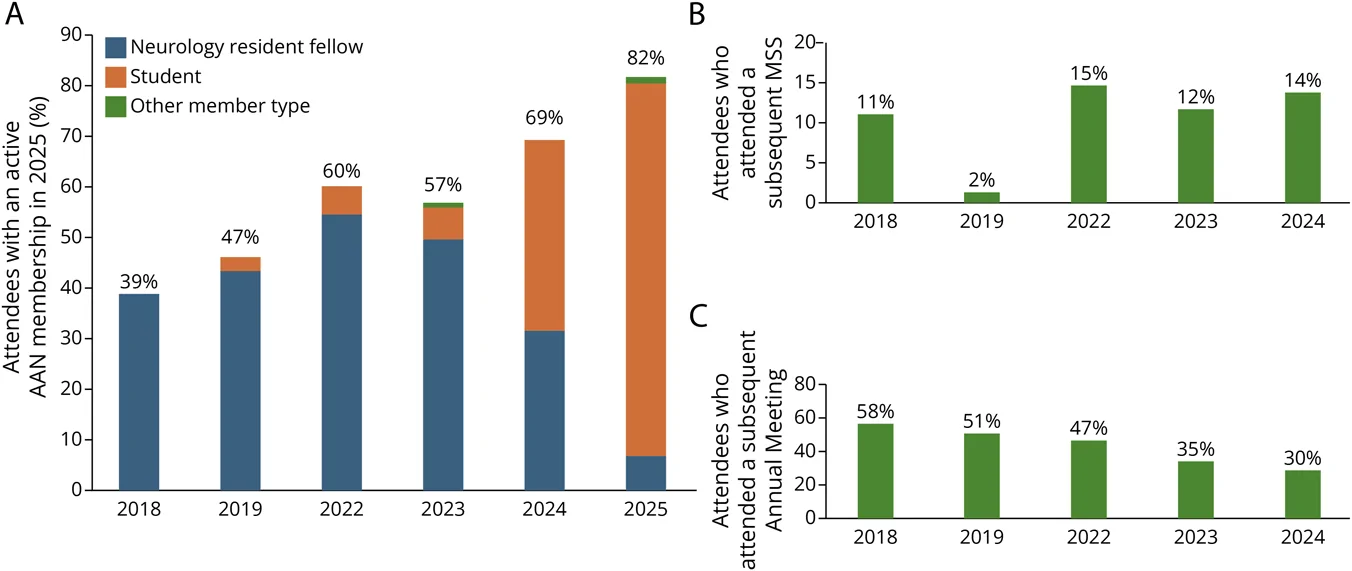
Attendee Engagement With the AAN in Subsequent Years (A) Active AAN membership in 2025. (B) Percent of attendees who attended a subsequent MSS. (C) Percent of attendees who attended a subsequent Annual Meeting. AAN = American Academy of Neurology; MSS = medical student symposium.

## Discussion

MSS participant survey results demonstrated that student participants valued learning about the different neurologic subspecialties and engaging in networking and mentorship opportunities, which reinforced their interest in a neurology career. These results suggest alignment with the original goals of the AAN Pipeline Subcommittee, which was charged with implementing strategies to expand the neurology pathway, primarily through the creation and maintenance of the MSS to foster interest in careers in neurology. Pathway initiatives for medical specialties generally operate across a spectrum of stages, from initial awareness and exposure to the field, through mentorship and professional identity formation, to ultimately influencing specialty selection. The MSS, as a half-day event during a national professional meeting, is positioned to influence the earlier stages of this spectrum: providing broad exposure to neurologic subspecialties, creating conditions for mentorship relationships to form, and clarifying a sense of professional identity among students already drawn to the field. The longitudinal survey results are best interpreted as indicators of these earlier-stage effects among self-selected participants, rather than as evidence of downstream workforce impact. The MSS in its earliest iterations incorporated sessions with hands-on demonstrations of medical devices used in neurology to improve broad exposure to the field and highlight innovations in neurology. Others have implemented similar approaches with a hands-on procedure workshop to stimulate interest in careers in clinical neuroscience.^6^ This type of programming in addition to the “Innovation Talks” offered as part of the MSS in 2021–2022, align with prior research that found that the second most important factor among medical students in a career choice in neurology was “intellectual content of specialty.”^13^

The survey results reflect attendee preferences for the subspecialty networking and welcome lunch components of the MSS, demonstrating the highest influence rankings, consistent with participants' own accounts in free-text responses. It is notable that the format of the symposium similarly shifted away from lecture-based content with greater time dedicated to networking opportunities such as the “Welcome lunch” and the Subspecialty “Networking session.” When asked how attending the symposium affected their career trajectory, participants most frequently described finding a mentor through the event and gaining a more personal and concrete understanding of neurologic subspecialties through direct conversation. This is consistent with prior work demonstrating that direct interactions with neurology providers may promote interest in careers in the field and that mentor-mentee relationships formed through such encounters sustain career commitment over time.^3,14^ Others have similarly found that the importance of role models is a key factor in influencing medical student specialty choices.^15^

Beyond the value that attendees found during the symposium, there is also potential value to mentors and volunteers in a sense of professional fulfillment in their support of fostering interest in neurology careers. In total, 36% of resident volunteers and 47% of faculty volunteers volunteered more than once, and their repeated volunteerism suggests that they found value in the experience. Although we do not have data on whether these interactions led to more longitudinal mentor-mentee relationships and did not survey volunteers about their experiences and perceptions directly, studies on the benefits of mentorship for mentors in enhancing personal and professional development may potentially explain the motivations for repeated involvement.^16,17^

Beyond the agenda and programming of the MSS, the longitudinal survey results indicated not only sustained interest in pursuing careers in neurology but also continued engagement with the AAN. We acknowledge that the population of study is skewed in that most respondents were already likely to pursue a career in either adult or child neurology. However, we hope that the efforts of the MSS not only sustained or enhanced this interest but also familiarized attendees with the opportunities at the Annual Meeting that led to continued involvement in the organization, as reflected by over 30%–40% of attendees from 2018 to 2019 who still had active AAN memberships in 2025. These longitudinal results are of value, as much of the existing literature on similar events to promote interest in a discipline have evaluation measures that are limited to more immediate, postsession measures of satisfaction.^4,6,9^

This study was limited in its sampling approach as the initial records of MSS attendee contacts primarily consisted of many now inactive institutional email addresses. However, more recently, the MSS has begun collecting both personal emails in addition to institutional emails to circumvent this issue in the future. Selection bias is another limitation inherent in the self-selected nature of MSS participants, most of whom had a preexisting interest in neurology. Response bias represents an additional and distinct limitation: Those who chose to complete the longitudinal survey may have been more likely to do so given particularly positive experiences with the MSS, potentially resulting in an overestimation of the degree to which the event reinforced career interest and sustained AAN engagement. The 12% response rate, while consistent with response rates in similar survey studies, further limits the generalizability of the longitudinal findings because the experiences and career trajectories of nonrespondents remain unknown. In addition, neurology trainees who attended the AAN annual meeting but did not attend the MSS were not surveyed to identify unique differences between those medical students going into neurology who attended the MSS compared with those who did not. This study also lacks a distinct comparison group as many of those who attended the MSS had a preexisting interest in a career in neurology. Therefore, this study is unable to assess the MSS's potential impact on medical students with neutral or little preexisting interest in neurology. Given that over 90% of longitudinal survey respondents had already intended to pursue a neurology specialty before attending, the data do not inform whether the MSS might influence students who are undecided or who consider themselves averse to the specialty. Engaging this population would be important to meaningfully expand the neurology pathway. Future studies should prospectively recruit students across the spectrum of interest, incorporate measures of specialty interest, and include a comparison group of students attending the Annual Meeting who did not participate in the MSS, to evaluate the impact of initiatives of this kind.

Future iterations of the MSS will continue to streamline the evaluation process to collect timely and relevant feedback from attendees on their engagement with the MSS and the value of the event. Feedback will continue to be incorporated to adapt the MSS agenda to the needs of medical student participants. As the field of neurology evolves in its various efforts pertaining to scientific research, advocacy, and global health, among others, we may find that student attendees may also change in their preferences for event programming and should remain adaptable to address these needs.

The experience of developing and iterating the MSS over 7 years offers several lessons that may be of value to clinician-educators seeking to promote interest in neurology or other medical specialties at their local institutions. The MSS rated most influential by participants were unstructured, relationship-centered events, ranking consistently above keynote speakers and panel discussions. When institutional resources are limited, investing in opportunities for direct student-faculty interaction is likely to yield greater impact than didactic programming. Second, the MSS is best understood as an intervention positioned to influence early-stage pathway outcomes: broad subspecialty exposure, formation of mentorship relationships, and development of professional identity as a neurologist. Third, nearly half of faculty volunteers and over a third of resident and fellow volunteers returned to participate in subsequent years, with common motivations including enjoyment of interactions with potential trainees, a desire to share their experience, and a sense of mentorship. Framing future opportunities around professional fulfillment may be effective. Fourth, the MSS experienced years of low postevent survey response rates until structural changes were implemented, including incentivizing completion through gift distribution and scholarship reimbursement contingent on evaluation submission. These changes markedly improved response rates and enabled evidence-based program adaptation; educators planning similar events should build evaluation incentives into the event design from the outset.

Based on these lessons, those interested in organizing similar, institutional versions of these events, such as in collaboration with their local Student Interest Group in Neurology (SIGN) chapters, may therefore choose to prioritize their efforts and resources toward opportunities for connection, networking, and mentorship. These events may also be particularly well-suited to engage students earlier in their training and potentially those not yet committed to neurology, given the lower barrier to access compared with a national meeting. Faculty participating in the MSS also found their efforts rewarding, which may be helpful in recruiting faculty locally. We also hope that the lessons learned in obtaining strong postevent evaluations are applicable to other educators as well and that they similarly remain flexible in responding to the local and institutional needs of students interested in neurology careers so that the neurology pathway can continue to remain robust for the years to come.

## Glossary

AAN: American Academy of Neurology
IMG: international medical graduate
MSS: medical student symposium
